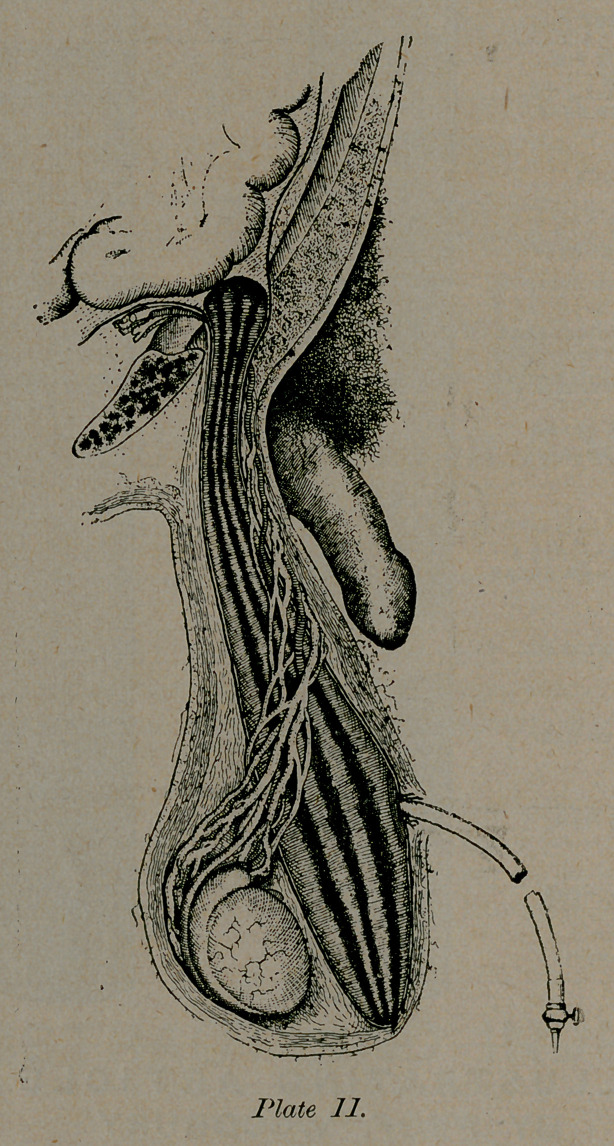# The Radical Cure of Hernia, Particularly with Children

**Published:** 1892-09

**Authors:** G. Felizet

**Affiliations:** Surgeon to Tenon Hospital, Paris, France


### Southern Medical Record.

A MONTHLY JOURNAL OF MEDICINE AND SURGERY.

VOL. XXII. ATLANTA, GA., SEPTEMBER, 1892. NO. 9

### Original Articles.

### THE RADICAL CURE OF HERNIA, PARTICULARLY WITH CHILDREN.

BY DR. G. FELIZET, SURGEON TO TENON HOSPITAL, PARIS, FRANCE.

Translated by THOS. H. MANLEY, A. M., M. D., Visiting Surgeon to Harlem Hospital, New York.

(*Continued.*)

4th Indication—To assure a perfect obliteration of the tract.

It is for this happy result that we must make a dissection minute—I do not say a laborious dissection, which has for its object the preservation of the sac, its serous and fibrous elements. M. Championniere has insisted, with reason, on the necessity of isolating the serous structures, which he considers as the ideal operation ; something difficult to realize.

Before him, M. C. B. Ball (*British Medical Journal*, 1884,) clearly described the rule for minuteness of execution, when publishing his procedure for radical cure, by tortion of the sac. The object in view is to secure the occlusion of the internal orifice, by isolating the serosa from the subjacent fascia. This idea was so natural, that long before

M. Ball, we have applied it as related by M. Legond, in 1883, and by M. Berjer, in the radical cure, after our kelotomies.

Our proceeding of tortion, which offers a surprising analogy with that of the Irish surgeon, consists in a slow twisting of the neck, through one of the folds of which a suture is carried, including the pillars on each side, and is lost from sight by being deeply imbedded in the loose, adjacent tissues.

In order to secure good results from tortion of the neck, without any harm to the serous funnel of the neck, it must be strictly isolated by a most minute dissection. That an operation for radical cure must embrace the destruction of the sac below the ligature or twist, is evident to our premising success on surgical logic; for interruption of the neck is effected and rendered permanent by the obliteration of this membrance of extreme tensity.

This delicate dissection which is indispensable always in this operation, even M. C. B. Ball himself, has not claimed priority for. We have deemed it necessary to particularly comment on the anatomical characters of this delicate membrane; how it should be ligated; how after section it quickly disappears within the abdomen, and the manner in which it responds to resistance.

We depend largely on the soft parts—the peripheral fibro-cellular tissues which have been isolated; but which have been carefully preserved, as representing an important, living mass, which blocks the tract and occludes the ring.

This mass serves an important purpose, but it, in itself, would be very insufficient if it were not for the irritation excited by the traumatism of operative interference, which swells, and thickens, and hardens it—which produces proliferation of cell elements, and causes adhesions with contractions, sufficiently solid, generally, for ordinary hernia; but especially those of children. But we believe that these adhesions and this contraction will not suffice in all cases.

Perhaps this hernial orifice is very large, and the tract very broad, or that the hernia is direct. The testes may have incompletely immigrated, being lodged above the ring, with a tendency to break through the new closure; either on one side, or through its centre. It is in this class of cases that we must have recourse to suturing the pillars of the ring, after the method of Mitchell Banks; but which is condemned by some surgeons, whose conclusions in this matter we do not fully agree with.

Let us see what may be accomplished by this operation of approximation of the rings by suture. We must not expect union of the two pillars, without freely refreshening their edges (a union of fibrous tissues is of little importance), with a view of effecting adhesions of an intimate character, similar to reunion of a wound; forming a sort of homogeneous wall, firm and permanent; the opening will be closed, and the pillars well fused. It might, however, be legitimate to insist on suturing the pillars of the ring, not for its definite occlusion, but for the purpose of narrowing its fibrous columns; though, after all, it is but a temporary occlusion of varying duration.

The union or support effected is shaped, organized and fixed where the pillars approximate, the cellulo-fibrous cement effused, maintaining a resistance which may occasionally contribute towards a radical cure.

In infantile surgery, suturing the pillars of the ring is often a necessary operation, when the testis is incompletely descended, and its maintenance externally is important. The case here related belonged to that class.

ECTOPIC TESTICLE, RIGHT SIDE—PHENOMENA OF STRANGULATED HERNIA—RADICAL CURE—SUTURE OF THE RINGS.

Eugene Baillard, 14 years old, entered the hospital January 27th, 1889, for treatment of ectopic testis and interstitial hernia; complaining of colicky pains and other symptoms of strangulation, at time of entrance. On January 29th, the operation for radical cure was performed. A transverse incision was made; the sac ligated and excised, and the pillars sutured. Retraction downwards of the testicle was difficult, but to secure its detention in the scrotum, the ring was closed. He recovered without incident in twelve days, but remained in the hospital two weeks, in order to accustom himself to the use of the truss, which was applied with a view of pressing the testis away from the abdominal aperture.

On March 27th, patient left hospital perfectly cured, without either colic or pain; the truss discarded. He returned to us eighteen months later, July 25th, 1890. The hernia was then totally cured. The testis is on a level with the external orifice. The pillars are *more widely separated than before the operation*, so that the testis might lodge in the interspace, if drawn into it.

We can easily comprehend why a truss, badly made and badly adjusted, may contribute towards the retention of the testicle and dilation of the pillars. The truss should often be cast aside altogether, in this complicated variety of hernia. The hernia is then cured by the gradual approximation of the fibrous walls, the testis unobstructed migrates downwards; the protrusion gradually disappearing.

An infant was sent to us September 4th. The cure was deceptive and incomplete; but since the suppression of the truss, which was more painful than useful, the rent between the pillars which lodged the testicle, had very considerably diminished.

### ECTOPIC TESTIS ON THE LEFT SIDE, COMPLICATED WITH INGUINAL HERNIA—RADICAL CURE.

Alfred Gras entered hospital Nov. 21st, 1889, for treatment of an external inguinal hernia, complicated with an undescended testis; the rupture is not troublesome, but the testicle is. During efforts at walking, the pains are atrocious. The right vaginal pouch was distended by serum, and contained a rudimentary testis attached to a shrivelled cord. The virility of this young man resided exclusively in the testicle on which we proposed to operate. According to the mother's testimony, this upward migration of the left testis was present but two months.

On the 23rd of November, the operation for radical cure was undertaken. The testis were found normal and exterior to the sac, which was very thin and delicate. The sac was so diminutive and fragile that it seemed impossible to ligate it. The testis was drawn downward, the intestine reduced, the pillars sutured with three strands of catgut. Result: Cure in one month. The incision did not unite by primary union, but partly re-opened and separated. He left the hospital January 12th, 1890.

The testicle is fixed against the abdominal wall, close to the sutured orifice. It is rather indurated, slightly immovable in the mass of cellular tissue in this region. He returned to us six months later with no evident tendency of the return of the hernia. The pillars of the ring, however, are separated and the testicle, although it is not possible to draw it downward, mounts readily and is lodged in the inguinal tract, which gapes widely outward. Suture of the ring in this case, as the proceeding did not produce coalescence of the fibrous structures; but nevertheless the operation has favored a cure, for without it the neck could not be ligated nor the sac excised.

### FOURTH OPERATION—HYDRATED CYST OF THE LIVER, COMPLICATED WITH CONGENITAL INGUINAL HERNIA ON RIGHT SIDE—RADICAL CURE WITH SUTURE OF THE PILLARS.

Earnest R., six years old, entered February 5th, 1889, for treatment of a large hydatid occupying the entire convex surface of the liver, and a large inguino-scrotal tumor on the right side, which gave evidences of threatening strangulation, by violent pains radiating to the lumbar region. A diagnosis of reducible inguinal hernia was made. It had attained the volume of a hen's egg. The ectopic gland was high up at the external ring. From February 5th to March 5th, treatment of the hydatid cyst was continued by puncture, aspiration and injection of Van Sweeten's liquid. Result: Cure without accident.

March 10th, operation for the radical cure was commenced. The neck of the sac was ligated with catgut The columns were approximated by three silk sutures, after the testicle was drawn towards the base of the scrotum. The testis was easily depressed and recognized as its dependent elevation was not attributable to shortening of the cord. Result: Very good.

This was a delicate child, with soft distensible abdominal walls and weak muscle. On July 8th, 1890, the child returned to know if he must continue the employment of the truss.

He had experienced no more pains and signs of strangulation since the operation. The testicle was now nearly as high as its congener. It rises and lodges in the aperture of the ring which it approaches, but does not become fixed there. There is now no hernial propulsion during effort of coughing. No truss. The infant comes to us July 18th, that we may see the consequences of laying the truss aside. The inguinal region is feeble on both sides, but one can feel on the side invaded a large projection responding to the general weakness of the girth. We advised, as a matter of prudence, that he wear a double bandage, and continue it until that age is attained when the natural forces will provide ample security.

The sutured pillars, although they did not effect definite occlusion, has permitted one to secure the radical cure, by the temporary adhesion of their margins, and the use of the truss has certainly facilitated the descent of the testes by a mechanism, indirect and curious. We would be disposed to convince ourselves that the narrowing of the ring has bound together the elements of the cord. The stricture of the cord has occasioned a veritable stasis of the mucous circulation—a species of experimental varicccele, as we have seen it during an operation for this condition, which results in increase in the weight of this organ, an elongation of the cord and finally a descent of the testis.

From the preceding review, it is evident that suture of the pillars serves as a temporary prop, which effects a useful purpose, until a radical cure is produced. Catgut and silk are not reliable. We now employ a very fine gold suture, which seems to secure a more permanent closure of the pillars. We have actually adopted two features of the *suture dorn*, and will if possible publish our results later.

5th Indication—To establish a plane of peritoneum, solid and strong, in place of the funnel of the internal orifice.

Such is the last indication. All this anatomical reposition would be wholly useless, if the parts were not most minutely adjusted, which we proceed to pass in review. This bottle-shaped infundibulum at the internal orifice may be replaced by a plane of peritoneum. This latter would suffice in restraining the protrusion if it were of sufficient strength and in proper position; if the ligature obliterated the pedicle of the sac; for it would seem that the peritoneum may resist the formation of a hernia, although a very delicate membrane.

It would represent a wrinkled surface; star-shaped, the rays of which would be from the centre of the ligature to the neck.

But for this neck to be properly ligated so as to constitute a solid resistent mass, it is necessary that the ligature include only the peritoneum. Here, the difficulty commences. To assure the perfect dissection of this serous membrane, we must commence low, as in the operation described by C. B. Ball, in his procedure for radical cure, by tortion of the neck; though, if we do a very fine dissection, there is danger that we may overstretch or lacerate it.

If we do not effect perfect isolation, we need not hope to be able to free and detach it from the cellular elements, and from the fascia. With the correct operative mode, a complete dissection is tedious, difficult and dangerous; while an incomplete dissection does not assure independence of the peritoneal membrane; but on the contrary, leads to adhesions in the inguinal tract, which may compromise the final success and cure. The procedure which we will describe, permits us, without loss of time, to meet the indications for radical cure which we have often realized.

Our description of the manual of operation will be brief.

We have, up to the present time, put the method to the test nearly twenty times, with adults and children; and we are indebted to it for a rapidity of execution, which is an important factor in the success of these operations in both extremes of life. We avoid a slow operation—the possible wounding of the vas deferens, the vessels and nerves—as we practice it to-day. An approximate operation, and insufficient, we have seen; as a result, imperfect obliteration of the tract without complete effacement of the internal orifice, or the formation of a permanant, resisting plane.

Unsatisfactory results often depend on difficulties of dissection; and these difficulties arise from the fact that the dissection is executed in a region in which the anatomical elements are found in such great disorder, that accidents from too free use of the scalpel, are possible. Now, these accidents are possible, because not only are the vessels and vas deferens displaced, but because in searching for them, we may occasion a movement which may do damage.

Raise and fix the field of operation so that this may be easily stripped, and above all, that the serous sac may be dissected as freely as the peritoneum, of which it is the continuation, without tediousness; without its escape—without lacerating it; isolating it in its integrity as easily and completely as a lipoma. Such is the aim we have in view, and such we think we have attained.

DESCRIPTION OF APPARATUS.

A balloon of red rubber-gauze, thin and suple, pear-formed, having a length of a little more than ten centimetres in its long axis, and a diameter of six centimetres, at the moment of inflation. This extends from the base upward, along the sides of the sac, and forms an uninterrupted tube, communicating with an inflating tube. Such is the simple device of M. Galaut, which we have employed for more than four years. This pear passes easily through the length of the pouch to the aperture, about twenty-five centimetres, and has a volume about equal to the index finger. The rubber is of a deep red color, which permits us by its transparence, to see layer by layer of the parts as exposed. As the air-bag is not only for filling and distending the hernial sac, but it also presses forward the entire inguinal tract, to the proportion of which it is indifferent, there is no difficulty of adjustment. An ovoid from eight to ten centimetres in its great axis, of which its greatest enlargement will be inferiorly, and of which the small extremity continues as a cylinder, reduced to three centimetres; destined to lodge in the inguinal tract, and obliterate it during operation.

The apparel which we employ for children, is very small, from four to six centimetres in its long axis, with diameters of the globe and extremity in porportion. It has the advantage in a certain class of cases, when we propose to push the dissection to, or beyond the pillars, not only at its dome, but on the side, to distend equally and simultaneously the entire sac without detriment. We have now definitely adopted this mode of proceeding, and produce the designs here.

DESCRIPTION OF THE RADICAL OPERATION FOR INGUINAL HERNIA.

Proper precautions having been taken, the patient is purged with a simple carthartic. He has taken in the evening a bath; has been shaved, if necessary. He is sounded with the catheter, to assure us that there is no part of the bladder in the contents of the sac.

*First Step*—Incision of the skin, commencing at that part nearest the ring, and terminating about the middle of the hernial globe. Ligation or tortion of the superficial arterioles. These are the only vessels requiring compression during the operation. The section may be made with a bistoury, a scissors or cannulated sound, through the natural fibrous planes, or adventitious formations. We have now come in close contact with the sac.

OPERATION FOR THE RADICAL CURE OF HERNIA.

Such is the first step of the operation, and it presents nothing new in particulars. The operation has been done of that variety depicted in Plate 1, the patient lying in his bed; and and as we have examined him lately, we have seen that the hernia has not descended, as the dissection was very delicate.

In an adult, the finger in the ring very easily appreciates the fibrous resistance and the free, sliding movement of the serous surfaces, the one on the other. But with a child, whose tissues are young and without much resistance, this perception is evident, though imperfectly so, and must be sought for with great prudence, when the bare remnant of the sac may be outlined. When the abdomen is distended, on the other hand, the task is easy.

*Second step of the operation*—It consists in establishing a small opening into the sac. If the sac is greatly distended, gentle taxis will return part of the intestine. The tension of the pocket being less, it is now easy to pinch it between the fingers and make a small aperture, four or five millimetres in length, by which we may easily perceive the bare intestine.

The forceps serve to fix the borders, and mark the orifice which is enlarged by the fingers.

(*To be continued.*)

## Figures and Tables

**Plate II. f1:**